# Unlocking Potential: The Re-emerging Role of Thyroid Hormone Therapy in Treatment-Resistant Depression

**DOI:** 10.1192/j.eurpsy.2025.855

**Published:** 2025-08-26

**Authors:** M. Cameira, P. Abreu, M. Pereira, M. Soares, I. Vidó, I. Lopes

**Affiliations:** 1 Hospital Júlio de Matos - ULS São José, Lisboa; 2 Hospital do Barreiro - ULS Arco Ribeirinho, Barreiro, Portugal

## Abstract

**Introduction:**

Treatment-resistant depression (TRD) poses a significant challenge in clinical psychiatry, affecting a substantial proportion of patients who do not respond adequately to standard antidepressant therapies. Recent studies have rekindled interest in thyroid hormone therapy as a potential augmentation strategy for enhancing treatment outcomes in both unipolar and bipolar depression. Thyroid hormones, particularly levothyroxine (LT4) and triiodothyronine (LT3), have been shown to influence mood regulation through their interactions with neurotransmitter systems, thereby offering a promising avenue for improving depressive symptoms in TRD patients.

**Objectives:**

This study explores the potential of thyroid hormones, specifically levothyroxine (LT4) and triiodothyronine (LT3), to enhance treatment outcomes for patients who have not adequately responded to standard antidepressant therapies. It also synthesizes recent findings on the safety profile of thyroid hormone therapy and examines potential gender differences in treatment response.

**Methods:**

A comprehensive review of the literature was conducted, focusing on studies published in the ten years that examined the role of thyroid hormones in TRD. Databases such as PubMed were searched for randomized controlled trials, meta-analyses, and observational studies that assessed the effects of LT3 and LT4 on depressive symptoms and treatment outcomes.

**Results:**

The literature review shows that adjunctive triiodothyronine (T3) significantly enhances treatment response in treatment-resistant depression. A randomized trial found that 53% of patients on T3 had a ≥50% improvement in depression scores after three weeks, compared to 19% for those on levothyroxine (LT4). While LT4 may benefit chronic cases, T3 is generally preferred for its superior efficacy. Thyroid hormone therapy also benefits bipolar depression, especially in rapid cycling patients. Functional imaging studies indicate that supraphysiologic LT4 can improve symptoms by modulating anterior limbic network activity. Some evidence suggests women may respond better than men. Overall, thyroid hormone therapy is well tolerated, with low incidences of adverse effects like tremor, anxiety, and palpitations. However, long-term efficacy remains mixed, with some studies showing no significant differences compared to placebo, and the safety of high-dose therapy is uncertain, highlighting the need for further research.

**Conclusions:**

Thyroid hormone therapy, particularly LT3 and LT4, is a promising augmentation strategy for treatment-resistant unipolar and bipolar depression. While LT3 is noted for its rapid response, further research is needed to clarify its long-term safety and optimal dosing. These findings highlight the importance of considering thyroid therapy for patients who do not achieve satisfactory outcomes with conventional antidepressants.

**Disclosure of Interest:**

None Declared

